# A Network Coding Based Hybrid ARQ Protocol for Underwater Acoustic Sensor Networks

**DOI:** 10.3390/s16091444

**Published:** 2016-09-07

**Authors:** Hao Wang, Shilian Wang, Eryang Zhang, Jianbin Zou

**Affiliations:** College of Electronic Science and Engineering, National University of Defense Technology, Changsha 410073, China; wanghao08@nudt.edu.cn (H.W.); zhangeryangnudt@163.com (E.Z.); zjb_nudt@163.com (J.Z.)

**Keywords:** ARQ, fountain codes, network coding, underwater acoustic sensor network

## Abstract

Underwater Acoustic Sensor Networks (UASNs) have attracted increasing interest in recent years due to their extensive commercial and military applications. However, the harsh underwater channel causes many challenges for the design of reliable underwater data transport protocol. In this paper, we propose an energy efficient data transport protocol based on network coding and hybrid automatic repeat request (NCHARQ) to ensure reliability, efficiency and availability in UASNs. Moreover, an adaptive window length estimation algorithm is designed to optimize the throughput and energy consumption tradeoff. The algorithm can adaptively change the code rate and can be insensitive to the environment change. Extensive simulations and analysis show that NCHARQ significantly reduces energy consumption with short end-to-end delay.

## 1. Introduction

The abundant resources in oceans motivate the increasing exploration activities towards the underwater environment. In addition, underwater acoustic sensor networks (UASNs) are increasingly attracting interest due to their wide applications such as environment monitoring, resource investigation, oceanography data collection, pollution monitoring and tactical surveillance [1,2,3,4,5]. All of these applications highly require reliable data transport techniques. However, underwater channels have unique features that are quite different from terrestrial wireless radio channels, so it is impossible to use terrestrial sensor network techniques in UASNs immediately.

Electromagnetic waves, optical waves and acoustic waves are the three main propagation media in UASNs. However, radio frequency (RF) waves suffer from high propagation loss and optical waves are affected by scattering and absorption in water. Thus, acoustic waves are preferred for long-range underwater communication, and this new kind of transmission medium brings some new features and challenges for the underwater reliable data transfer protocol design.

Underwater acoustic channels are affected by many factors such as path loss, noise, multi-path and Doppler spread. All of these factors cause high error probability and packet loss in acoustic channels. Moreover, the available bandwidth of underwater acoustic channels is limited and depends on both transmission range and frequency.

Another prominent feature of underwater acoustic channels is the long end-to-end delay caused by the low propagation speed of acoustic signals in water. The propagation speed (about 1500 m/s) of sound in water is five orders of magnitude lower than the radio propagation speed ($3\times 10^{8}$ m/s). Thus, the propagation delay is much longer than transmission delay and must be taken into consideration in the underwater data transfer protocol design.

In addition, most sensors in UASNs are not fixed in place due to the movement of water current. Empirical observation suggests that underwater objects can move passively at speeds of 1–3 m/s with water currents. The water temperature and some other factors can also affect the sound ray, which suggests that the code rate of the underwater system should be adaptively changed to meet the dynamic underwater environments. In such harsh network scenarios, it is very challenging to provide reliable and energy efficient data transfer in UASNs.

Several techniques have been proposed to achieve reliable data transfer in UASNs. Traditionally, researchers utilize feedback based techniques such as automatic repeat request (ARQ) [6,7,8] to improve the reliability in underwater environment. However, the large number of retransmission times caused by high packet error rate makes these protocols inefficient and the sender has to send redundant data packets that might have been received successfully by the receiver if their acknowledgements (ACKs) had been lost during transmissions. The low channel utilization limits the practical applications of such techniques.

Another class of existing protocol is based on error recovery technique. Forward error correction (FEC) based protocols might reduce the delay because they need no ACK or negative acknowledgment (NACK) from the receivers. In [9], the author proposes an adaptive redundancy transport protocol which applies BCH (Bose – Chaudhuri - Hocquenghem) coding to data transfer according to the distance between nodes. In [10], the author applies network coding to UASNs for reliable data transfer and demonstrates the efficiency of the scheme. A Multiple Paths and Network Coding (MPNC) [11] protocol is proposed to further improve the throughput and reduce transmission overhead of network. The network coding based protocol uses three disjoint paths and transmits XOR (exclusive or) packets in the middle path. The performance of MPNC can be further improved by reducing the paths as in [12], where a twin path and network coding (TPNC) protocol is proposed to achieve lower energy consumption. However, a fatal weakness of such schemes is that they need a large number of redundant check packets to achieve high reliability as these schemes lack the retransmission process and feedback information from the receiver. The drawback is extremely obvious when data packets are transmitted hop by hop for most UASN applications, which means that each hop needs a larger number of transmission redundancies to ensure the reliability of the whole network. Thus, they are not preferred for most battery-powered devices in UASNs. In addition, without the feedback information from the receiver, these protocols usually have fixed parameters like code efficiency and cannot adapt to the dynamic channel in UASNs.

Recently, a class of hybrid ARQ (HARQ) protocols that combine FEC and ARQ is attracting increasing interests and shows better performance in UASNs. Some erasure code based protocols like Segmented Data Reliable Transport (SDRT) [13,14], Fountain Code based Adaptive multi-hop Reliable data transfer (FOCAR) [15] and Practical Coding based Multi-hop Reliable Data Transfer (PCMRDT) [16] utilize erasure code to transmit packets in blocks, thus reducing the usage of ACKs or NACKs. However, the receivers in these protocols only receive packets from their adjacent previous nodes and do not fully exploit the broadcast property in UASNs. A Network Coding with Implicit Acknowledgement (NCIA) protocol [17] is proposed to exploit the spatial diversity in UASNs. The nodes in NCIA overhear implicit ACK packets from the whole network if they are in range but data are sent by packets and they still need one feedback packet for each successfully received packet and thus have a significant end-to-end delay. In [3], the author proposes a cooperative hybrid ARQ (CHARQ) protocol that combines cooperative ARQ with incremental redundancy-hybrid ARQ. This protocol utilizes multiple relays to increase the system throughput, but it is still inefficient as the relay sets often do not obtain enough packets from the sender in the high packet error rate channel and many reduplicative check packets are sent. In a word, the previous HARQ protocols achieve low channel utilization and perform inefficient. In addition, they lack an adaptive code rate estimation for dynamic underwater channel to ensure the network can work at its optimal achievable performance.

In this paper, we propose a novel network coding based hybrid ARQ (NCHARQ) protocol to promote the performance of data transfer in UASNs. The nodes in the network can overhear packets from all nodes in the network and packets are transmitted by blocks to reduce the redundant ACKs and NACKs. In addition, an adaptive sending window estimation algorithm is designed to ensure that the system could work at its optimal achievable performance in the dynamic underwater environment. The main contributions of this paper can be summarized as follows:

(1) We propose a reliable data transfer protocol for UASNs based on network coding and HARQ that can achieve higher throughput performance and reduce the energy consumption. We use an improved variant of fountain code with high efficiency and the relay nodes can encode and decode quickly.
(2) To reduce the redundant feedback, we design a block-based transmission scheme. The encoded packets are gathered in blocks and a selective-repeat ARQ scheme is utilized to ensure the successful transmission of each block. We also design an adaptive sending window length estimation algorithm to optimize the throughput, which can be adapted to the dynamic underwater environment.
(3) We design a dynamic 3D underwater network model and conduct extensive simulations to verify the outstanding performance of our proposed protocol in average delay and energy efficiency. The results show that our proposed protocol outperforms previous works, including stop and wait ARQ (S&W ARQ), SDRT and FOCAR. We also present a detailed analysis of the simulations.

The rest of the paper is organized as follows. We present the network model in Section 2 and give a brief introduction to the underwater channel model. We give a brief review of network coding and introduce a practical design of network coding based on fountain codes in Section 3. Then, we elaborate the process of our proposed protocol NCHARQ in Section 4, which is the main body of our paper. After that, we conduct the simulations and analyze the performance of different protocols in Section 5. Finally, we give a conclusion and suggest some future works in Section 6.

## 2. Network and Channel Model

In this section, we first introduce our target multi-hop underwater acoustic sensor network model and then present an algorithm to estimate the packet loss rate under the underwater acoustic channel.

### 2.1. Network Model

**Figure 1:**
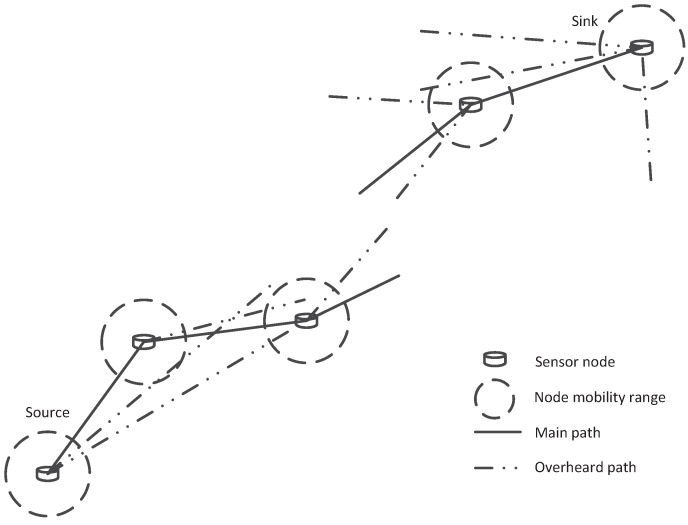
An illustration of network model for underwater acoustic sensor networks.

The network model of underwater acoustic sensor networks is illustrated in Figure 1. We consider a deep water environment for data transfer here. The source sensor node collects information in the underwater environment and sends data packets to the sink node hop by hop. Several relay nodes are distributed randomly between source node and sink node and anchored to the ocean bottom. There exists a main multi-hop path and data packets are transmitted mainly through this path. The path can be obtained by a suitable routing process and the routing process is not the concerned issue in this paper. Each node broadcasts its encoded packets to the whole network. All the receiving nodes can not only receive packets from its adjacent main path node, but also overheard packets from all the nodes in the network. We mainly focus on the reliable data transfer protocol on these paths. In addition, as most of the underwater sensors can not have a fixed position in the moving underwater scene, we suppose the nodes can appear at any position within its mobility range (the dashed circle with radius *R*) in our network model. The total number of nodes is N+1. Data packets are generated and encoded at node 0 (source node) and transmitted to the destination node *N* (sink) through N-1 relay nodes. The distance between node *i* and node *j* is defined as $d_{i,j}$. Then, the following condition holds for all nodes in the network:

(1) $$d_{i,j}>d_{i,i+1}\;\;,\;\;for\;all\;\left|i-j\right|>1.$$

Equation (1) means that for node *i*, the adjacent relay nodes in the main multi-hop path always have less distance than the nodes in the overhearing path and thus have a better channel condition. This condition is always true for a suitable routing protocol.

### 2.2. Underwater Channel Model

We present a brief introduction to the underwater channel and propose an empirical method to estimate the packet error rate between any two nodes according to the underwater channel. The acoustic transmission in underwater channel is affected by three main factors: path loss, ambient noise and fading [18]. For a given distance *d* and frequency *f*, the path loss for sound propagation $TL\left(d,f\right)$ is described by Urick [19] and can be expressed as:

(2) $$TL\left(d,f\right)=A_{0}d^{k}a{\left(f\right)}^{d}$$

where $A_{0}$ is a normalizing parameter. *k* is the spread factor and k=1.5 for practical spreading. $a\left(f\right)$ denotes the sound wave absorption loss and is modeled by the Thorps formula [20] as:

(3) $$10\log a\left(f\right)=\frac{0.11f^{2}}{1+f^{2}}+\frac{44f^{2}}{4100+f^{2}}+\frac{2.75f^{2}}{10^{4}}+0.003\;\;\left[dB/km\right]$$

The ambient noise in underwater environment is affected by several factors. According to the famous Wenz noise model [21], there are four main noise sources in the ocean: turbulence $N_{t}$, shipping $N_{s}$, waves $N_{w}$ and thermal noise $N_{th}$. The total power spectral density of noise level NL(f) for a given frequency *f* is the accumulation of these factors as follows:

(4) $$NL\left(f\right)=N_{t}\left(f\right)+N_{s}\left(f\right)+N_{w}\left(f\right)+N_{th}\left(f\right)$$

where

(5) $$\begin{array}{c} 10\log N_{t}\left(f\right)=17-30\log f,\\ 10\log N_{s}\left(f\right)=40+20\left(s-0.5\right)+26\log f-60\log\left(f+0.03\right),\\ 10\log N_{w}\left(f\right)=50+7.5w^{1/2}+20\log f-40\log\left(f+0.4\right),\\ 10\log N_{th}\left(f\right)=-15+20\log f, \end{array}$$

where *s* is the shipping activity factor that takes values between 0 and 1 for low and high activity, respectively. *w* is the wind velocity in m/s. Then, we can obtain the SNR (signal-to-noise ratio) per bit $\gamma_{b}$ from passive sonar equation described in [19]:

(6) $$\gamma_{b}=SL-TL-NL+DI$$

where SL is the source level and DI denotes the directivity factor. For omnidirectional hydrophones, DI=0. TL and NL come from Equations (2) and (4), respectively. If we use binary phase shift keying and suppose Rayleigh fading for the underwater multi-path channel as many researchers do [9,18,22,23], the average bit error rate (BER) $P_{b}\left(i,j\right)$ between node *i* and node *j* can be obtained [24] as follows:

(7) $$P_{b}\left(i,j\right)=\frac{1}{2}\left(1-\sqrt{\frac{{\bar{\gamma }}_{s}\left(i,j\right)}{1+{\bar{\gamma }}_{s}\left(i,j\right)}}\right)$$

where

(8) $${\bar{\gamma }}_{s}=10^{\gamma_{b}/10}$$

Thus, for a packet with *L* bits, the packet error rate $P_{pac}\left(i,j\right)$ between node *i* and node *j* can be obtained as follows:

(9) $$P_{pac}\left(i,j\right)=1-{\left(1-P_{b}\left(i,j\right)\right)}^{L}$$

Then, for a given source level SL and distance $d_{i,j}$, we can obtain its packet loss rate from Equations (6), (7) and (9).

## 3. Network Coding Design

### 3.1. Review of Network Coding

Network coding was first introduced by Ahlswede et al. [25] to improve the throughput and capacity of the network. In network coding, the nodes in the network can combine the received data in a linear or non-linear process and each node is able to receive the combined data packets from different nodes. It has been proved by Li et al. [26] that linear network coding can achieve the max flow bound on information transmission. Fragouli et al. [27] present an instant primer of network coding that can achieve near maximum throughput without the knowledge of network topology. However, traditional network coding is not suitable in our proposed protocol for the computation complexity: the decoding process of random linear coding needs matrix inversion, and the complexity is $O\left(K^{3}\right)$ for *K* data packets. It will be very complex for a large number of original packets. The complexity is not practical for real-time decoding at each node in the sensor network. To solve this issue, we propose a novel network coding scheme based on fountain code in this paper.

### 3.2. A Practical Network Coding Based on Fountain Code

**Figure 2:**
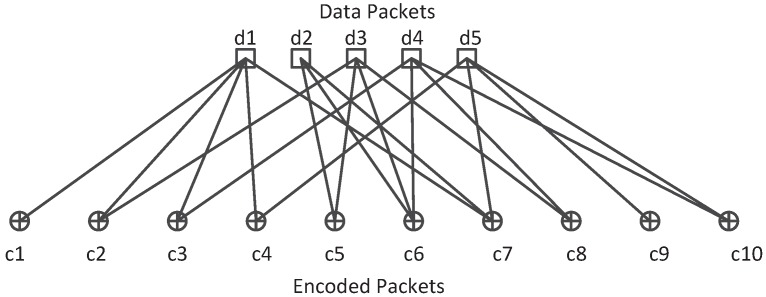
A typical encoding process of LT codes.

Fountain code is a class of erasure code and used for recovering lost packets in the erasure channel. The representative fountain codes are Luby transform (LT) codes [28] and Raptor codes by Shokrollahi et al. [29]. The basic idea of fountain codes can be described as follows : the sender divides the source file into *K* packets and encodes these packets into *N* packets (N≫K), anyone who wishes to receive the source data file collects the encoded packets until the number of received packets is a little larger than *K*, then the receiver can recover the original file with these packets. The advantage of fountain code scheme in a multicast system is that any node in the network can recover the original data packets with enough coded packets and the decoding process could be done in a linear time with high successful probability [28]. In addition, if each node encodes the data packets independently and randomly, the receiver can recover the original data packets with encoded packets from different sending nodes. In this way, the fountain code shares the same properties with network coding and can be treated as a particular class of network coding. A typical encoding process of LT codes is illustrated in Figure 2. There are 5 original data packets and 10 encoded packets in the Tanner graph. Each output encoded packet is the XOR of randomly selecting data packets. The number of original packets participating in an encoded packet relies on the design of output degree distribution $\rho \left(d\right)$. The decoding process of such codes can be performed in a simple way by belief propagation (BP) algorithm [30] as follows:

(1) Find a coded packet $c_{k}$ that is connected only to one data packet $d_{j}$ and then $d_{j}$ can be decoded by $d_{j}=c_{k}$. Remove all the edges connected to packet $d_{j}$.
(2) Repeat (1) until all the data packets are decoded or there is no existing packet that is connected to only one data packet.

The design of degree distribution $\rho \left(d\right)$ is crucial for the coding efficiency. However, most $\rho \left(d\right)$ designs like robust soliton distribution [28] require a large number of redundant packets and are optimized for large file transmission over high bandwidth links. We prefer a simple degree distribution of SVT (simple variant of tornado) codes by Xie et al. [13] in our protocol design. The SVT codes can only produce a limited number of encoded packets, but they can achieve higher coding efficiency. It is sufficient in our design because of the limited channel bandwidth in the sparse underwater acoustic sensor networks. Each node generates the encoded packets independently with the same output degree distribution $\rho \left(d\right)$. The encoding vector could be sent as part of the packet header overhead. In this way, each node in the network can recover the original data packets successfully if the node can receive a sufficient number of packets from any nodes in the network. Thus, the information in the network can be used sufficiently and an optimized performance could be achieved by designing an efficient data transfer protocol.

## 4. Protocol Design

The key idea of our proposed NCHARQ is to receive encoded packets from all nodes in the network and thus the system can obtain a space diversity gain. We choose to send packets block by block and hop by hop. There are two aspects for the reason that we choose to transmit data in such mode:

(1) The propagation delay is much longer than transmission delay in underwater channel. Thus, it will be very inefficient to inform the sender of the ACK or NACK information of every packet, and the channel utilization would be very low due to the long end-to-end delay. The packet error rate will be much lower and a higher throughput can be achieved with the help of relays.
(2) In a packet by packet transmission mode, if a node sends a packet to a receiver successfully but the sender could not receive a valid ACK from the receiver due to the high packet error rate, the sender has to send the packet again and the duplicated information will be useless for the receiver. For example, if a packet is transmitted successfully from node *i* to node i+3, it will be wasteful for node i+1 and node i+2 to send the packet again. However, due to the high packet error rate and half-duplex system in UASNs, it would be hard for all of the nodes between node *i* to node i+3 to receive the valid ACK from node i+3. Thus, useless retransmissions will cost high energy and bring a long end-to-end delay.

In a block-by-block transmission mode, the receiver may only send a minimum of one ACK packet back for several packets in a block and thus reduce much propagation delay. In addition, although a single packet may be able to skip hops in its transmission process, the probability is very low that all the packets in a block can skip hops and reach a node through fewer hops. Thus, any node in the network can simply send ACK packets to its adjacent upstream node and thus reduce the useless retransmissions caused by long-distance ACK packets transmission failure. To achieve the max throughput, we also have to control the sending window size precisely. In this section, we first present a detailed introduction to our proposed NCHARQ protocol and then design an adaptive sending window size control algorithm.

### 4.1. Protocol Overview

**Figure 3:**
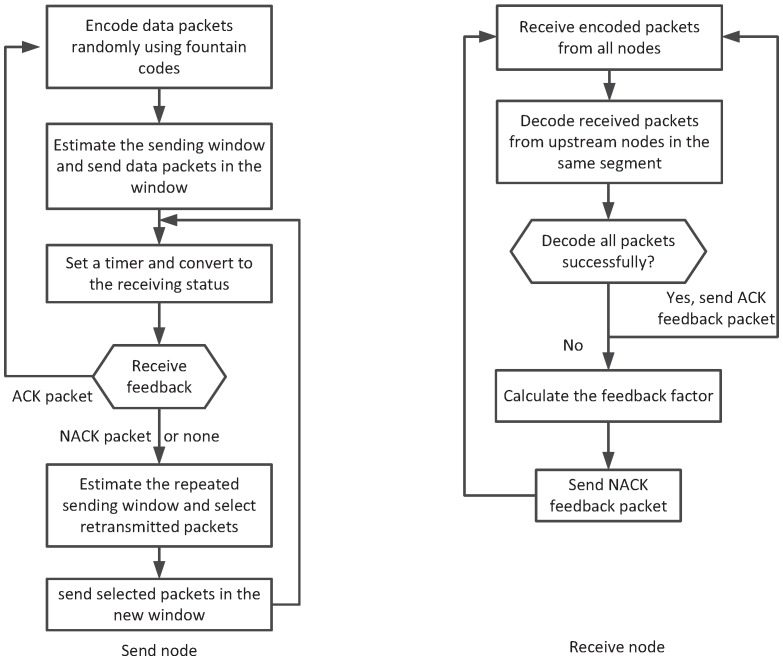
The overall process of network coding based hybrid ARQ (NCHARQ) protocol.

The overall process of NCHARQ protocol is illustrated in Figure 3. In NCHARQ, the source node first groups the original data packets into blocks, and each block contains *K* data packets. The original data packets *K* in a block size is set as a system parameter. The packets are sent hop by hop and block by block in the network. The sending nodes encode each block into *N* encoded packets with fountain code independently. Then, $K_{i}^{\prime }\left(K<K_{i}^{\prime }<N\right)$ out of *N* encoded packets are selected randomly according to the window size estimation algorithm in node *i*, and the sender sends the $K_{i}^{\prime }$ encoded packets at one time. Then, it waits for the feedback from the adjacent downstream node to decide whether to send a new block or retransmit part of encoded packets in the previous block. The process of NCHARQ in sender and receiver is described in Table 1.

Clearly, there are two main problems that remain unsolved. One is how to choose an optimal $K_{i}^{\prime }$ so that each node can ensure high probability of data successful recovery while decreasing the number of retransmissions. As the propagation delay is much longer than transmission delay in UASNs, the delay for retransmission will largely decrease the channel utilization and result in a large end-to-end delay. The other problem is how to select the *m* encoded packets (including the number of packets and the selection of packets) when the retransmission process is needed. Traditional protocols like SDRT [13] and FOCAR [15] present some offline algorithms to estimate optimal sending window size; however, these algorithms cannot adapt to the dynamic environment in the underwater channel. Here, we present a practical way to estimate $K_{i}^{\prime }$ and *m* efficiently.

### 4.2. Window Size Estimation

#### 4.2.1. Block Size Estimation

We propose a practical algorithm to estimate the optimized $K_{i}^{\prime }$ based on a digital second order loop filter. The advantage of loop-filter is that we can achieve real-time tracking control while reducing the effect of instantaneous channel varying. We can obtain the input calibration factor $\Delta n_{i}^{\prime }\left(k\right)$ of the digital filter from Equation (10):

(10) $$\Delta n_{i}^{\prime }\left(k\right)=\left\{\begin{array}{c} \alpha K-N_{r,i+1}\left(k\right),\;\;\;if\;\;ACK\;\;or\;\;NACK\\ 0,\;\;\;\;\;\;\;\;\;\;\;\;\;\;\;\;\;\;\;\;\;\;\;\;\;if\;\;none \end{array}\right.$$

where *K* is the number of original data packets in a block, *α* is the redundant factor and αK represents the number of encoded packets that a receiver needs for successful decoding on average. For the fountain code we used here, we can set α=1.2 empirically as explained in Section 5.4. $N_{r,i+1}\left(k\right)$ is the number of encoded packets actually received at node i+1 in the network for the *k*th block, and $N_{r,i+1}\left(k\right)$ is sent together with ACK and NACK packets for window size estimation. $\Delta n_{i}^{\prime }\left(k\right)$ will be a negative number mostly when an ACK packet is received, which indicates that we could reduce the sending of redundant encoded packets and vice versa. Thus, we can update the sending block size according to Equation (11):

(11) $$\begin{array}{c} K_{i}^{\prime }\left(k+1\right)=K_{i}^{\prime }\left(k\right)+Ki\times A_{c}\left(k\right)+Kp\times \Delta n_{i}^{\prime }\left(k\right)\\ A_{c}\left(k\right)=\sum_{j=1}^{k}\Delta n_{i}^{\prime }\left(j\right) \end{array}$$

where $\Delta n_{i}^{\prime }\left(k\right)$ is the input variable obtained from Equation (10). $A_{c}\left(k\right)$ is the cumulative factor and represents the impact of slowly varying environment. Ki and Kp are the impact factors that represent the influence of statistic and transient change of the underwater channel, respectively. Then, $K_{i}^{\prime }\left(k+1\right)$ is the sending window size estimation at node *i* for sending the (k+1)th block. With the initial value $K_{i}^{\prime }\left(1\right)=\alpha K$ and $A_{c}\left(1\right)=0$ for the first block, we can obtain the asymptotic optimal estimate of the sending block window size $K_{i}^{\prime }$ for node *i*.

#### 4.2.2. Retransmission Window Size Estimation

In fact, we cannot always guarantee a 100% success ratio for data recovery at one block sending round. Although we could increase the overhead to raise the data transfer reliability, it is not practical for battery-powered underwater facilities. Thus, automatic repeat request (ARQ) is a promising technique to achieve high data transfer reliability in underwater scenes. In traditional fountain code based hybrid ARQ protocols, such as SDRT [13] and FOCAR [15], retransmitted packets are randomly selected from the encoded packets in a block. This scheme is very inefficient because the retransmitted packets may have already been recovered with the previous transmitted packets and the sender has to wait for the NACK packet again and transmit more packets until some useful encoded packets are received successfully. It is particularly obvious when the output degree is low in the design of fountain code. Thus, we need an efficient scheme to ensure precise retransmission. In this paper, we propose a practical algorithm to estimate the retransmission window size. We divide the retransmission process into two situations. When $N_{r,i+1}\left(k\right)<K$, it means the receiver does not obtain a big enough number of encoded packets and the corresponding sender needs to send more encoded packets. The number of retransmitted packets $N_{s,i}\left(k\right)$ is obtained by Equation (12):

(12) $$N_{s,i}\left(k\right)=\left\lceil \frac{1}{1-p_{i}\left(k\right)}\left(K-N_{r,i+1}\left(k\right)\right)\right\rceil$$

where $p_{i}\left(k\right)$ is the packet error rate in the *i*th hop when sending the *k*th block and an estimation of $p_{i}\left(k\right)$ can be calculated as follows:

(13) $$p_{i}\left(k\right)=1-\frac{\alpha K}{K_{i}^{\prime }\left(k\right)}$$

However, when $N_{r,i+1}\left(k\right)\geq n$, it means the receiver has obtained enough number of encoded packets, but some of the packets may be reduplicative to some extent. Thus, we need to send a packet which is not recovered by the receiver yet, and this can be done by sending back the index number of the lost data packet with a NACK packet. We should note that the data packets are also part of the encoded packets in the design of fountain code. Thus, the retransmission process is summarized as follows: the sender reads the information of the NACK packet and sends $m=\max\left(N_{s,i}\left(k\right),1\right)$ packets to its corresponding downstream node. The retransmitted packets contain a lost data packet informed by the receiver.

## 5. Performance Evaluation

In this section, we analyze the performance of the proposed protocol with numerical simulations. The network model and channel model we used here is presented in Section 2.

### 5.1. Metrics and Simulation Parameters

We first define some metrics to measure the performance of NCHARQ.

(1) Transmission Redundancy : the metric is defined as in Equation (14):
  (14) $$\begin{array}{c} \eta =\frac{number\;of\;sent\;packets\;in\;all\;nodes}{number\;of\;original\;data\;packets} \end{array}$$
  This metric indicates the average sending packets for transmitting a data packet successfully and η≥1. A large *η* means the system is inefficient and thus more energy consumption is needed. The main source of energy consumption for underwater applications comes from the sending data process, and we mainly measure the energy consumption by calculating the number of sending packets under the same transmitting sound level. The larger the number of average sending packets for transmitting one data packet successfully, the more energy that the system consumes.
(2) Average Delay: the metric is defined as in Equation (15):
  (15) $$\begin{array}{c} \phi =\frac{time\;delay\;of\;sending\;all\;the\;packets}{number\;of\;original\;data\;packets} \end{array}$$
  This metric indicates the average end-to-end delay from the sender to the destination node for a single data packet transmission. A small *ϕ* means the system has a high throughput and better channel utilization.

The underwater acoustic channel is modelled by Matlab as a binary erasure channel (BEC). The relevant specifications and parameters are presented in Table 2.

**Figure 4:**
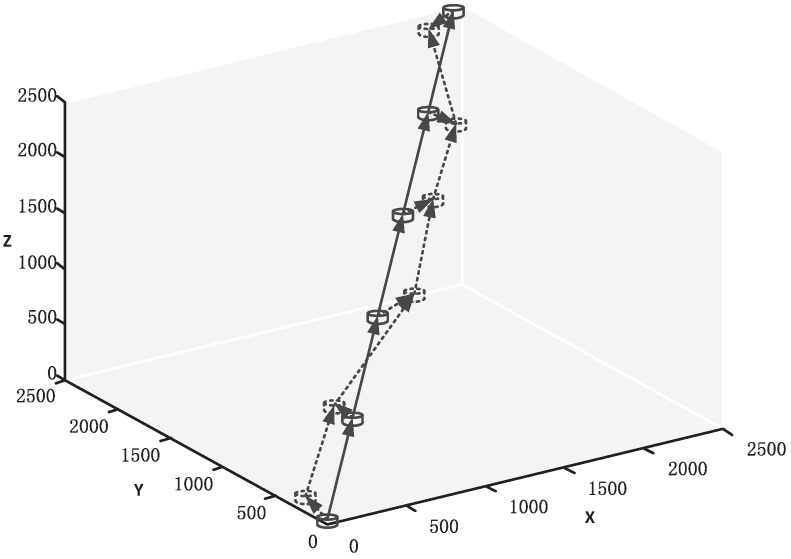
An example of six-node distribution.

We assume the nodes are distributed in a cubic box of 2500 × 2500 × 2500 $m^{3}$ as shown in Figure 4. At the initial state (time 0), Node 0 (source node) is located at (0, 0, 0) and Node *N* (destination node) is located at (2500, 2500, 2500). Thus, the total transmission range is about 4.33 km. The relay nodes are distributed uniformly in a straight line between Node 0 and Node *N*. All the nodes in the network can move randomly within their mobility range. Thus, the packet error rate $P_{pac}$ changes randomly with time passing by. We choose a relatively small data packet size (50 Bytes) and original data packet number (100) in one block here, so our parameters can adapt to a high-dynamic underwater environment.

### 5.2. Optimum Window Size

In this section, we present the numerical simulation proof for the window size estimation algorithm proposed in Section 4.2. To verify the validity of the proposed algorithm more clearly, we firstly conduct simulations on a simple statical network model. All nodes in the network are fixed in place and each adjacent node pair has the same distance of 866 m. The transmitting sound level is fixed to 124 dB. The total number of nodes is six, Node 0 is the source node and Node 5 is the destination node. All the residual nodes act as relays. The packet error rate can be obtained from Equation (9) as discussed in Section 2.2. Particularly, the packet error rate vector is $P_{pac}$ = [0.2276 0.6021 0.8810 0.9835 0.9992] for sending packets from node 0 to nodes 1–5. Although some nodes are far apart from each other, they still have the opportunity to receive the encoded packets at a relatively high packet error rate.

**Figure 5:**
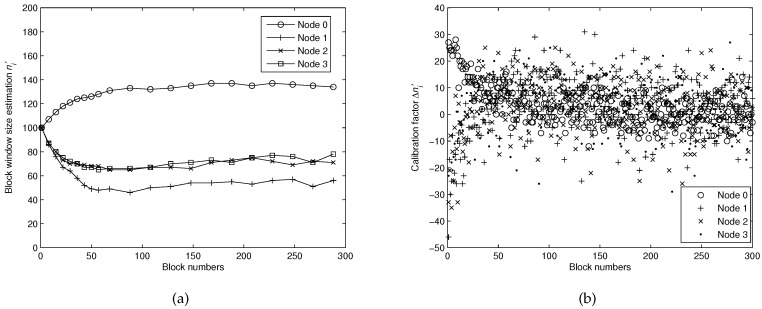
(**a**) the convergence procedure of estimating the block window size $n_{i}^{\prime }$ for different nodes; (**b**) the calibration factor $\Delta {n^{\prime }}_{i}$ for different nodes.

Figure 5a shows the convergence procedure of estimating the optimized sending block window size. To combat the high packet loss rate in an underwater environment, the source Node 0 has to send more encoded packets to ensure high probability of successful decoding at Node 1. Although the number of original data packets is 100, we need to send about 135 encoded packets to ensure that Node 1 can recover the original data packets with high probability. However, for Nodes 1–4, the number of sending encoded packets can be largely reduced with the help of node cooperation. For example, Node 2 can receive encoded packets from both Nodes 0 and 1. Thus, when it is the time slot for Node 2 to receive packets from Node 1, Node 1 can just transmit about 53 encoded packets, and it is sufficient for successful decoding at Node 2. However, it shows that Nodes 2 and 3 need to send about 71 packets, which is higher than Node 1. This is because Node 1 sends much less packets than Node 0, so Nodes 2 and 3 will receive less packets from their upstream nodes. All the estimation window size will become almost constant after sending 50 blocks, which means the proposed algorithm can optimize the window size within a short time. Figure 5b shows the calibration factor $\Delta n_{i}^{\prime }$ factor at each node. The definition $\Delta n_{i}^{\prime }$ can be referred to Section 4.2.1 and the change of $\Delta n_{i}^{\prime }$ can represent the dynamic variation of lost packets. It is shown that, although the packet loss rate is fixed, the number of lost packets is randomly distributed at each block sending round. Thus, it is not wise to estimate the block sending window with only one feedback packet. With the help of digital loop filter, the estimated window size can still stay steady with instantaneous variable $\Delta n_{i}^{\prime }$.

**Figure 6:**
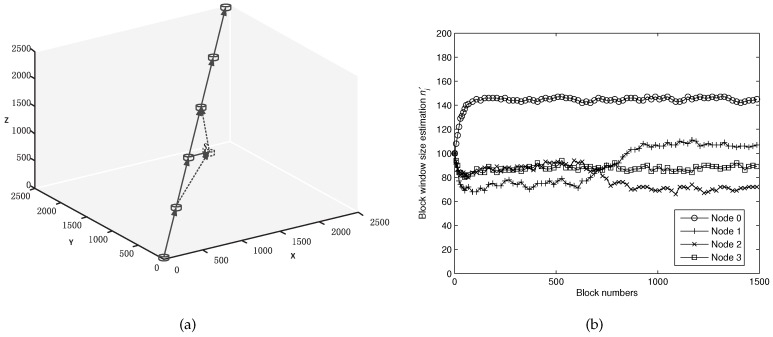
(**a**) an illustration for movement path; (**b**) the convergence procedure of estimating the block window size for moving nodes.

Next, we conduct some simulations on mobile nodes to verify the validity of the proposed algorithm. The movement network model is illustrated in Figure 6a. All the nodes in the network still remain when they are sending the 0 ∼ 600th blocks. Then, the position coordinate of Node 2 begins to move by step (1, 0, 0) at each block sending round and still remains again after moving 300 m. Figure 6b shows the estimated block sending window size for 1500 blocks. It can be seen clearly that the estimated window changes rapidly at block numbers from 600 to 900. As Node 2 moves far away from Node 1 and close to Node 3, the packet error rate $P_{pac}\left(1,2\right)$ increases while $P_{pac}\left(2,3\right)$ begins to decrease. Thus, Node 1 needs to send more more packets and Node 2 can reduce the number of sending packets. The estimation becomes almost constant again after the movement ends. From the above simulations, we can conclude that the proposed window size estimation algorithm works well for both statical and mobile nodes. We can trace the changes of packet error rate in the underwater channel and update the optimized window size accordingly.

### 5.3. Effects of Transmission Power, Number of Hops and Redundancy

**Figure 7:**
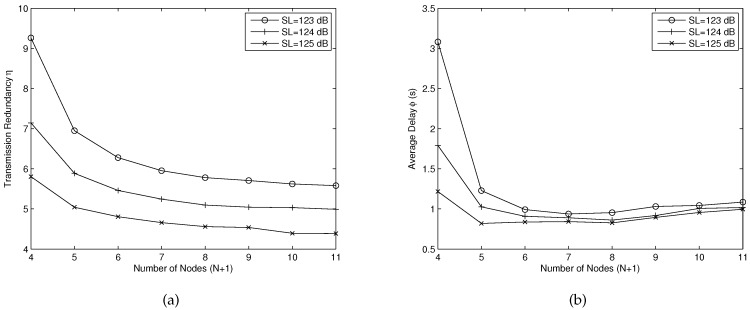
(**a**) the transmission redundancy *η* for different transmitting sound level SL; (**b**) the average delay *ϕ* for different transmitting sound level SL.

Figure 7 shows the transmission redundancy *η* and average delay *ϕ* for different transmitting sound level SL. In this simulation, we assume all nodes are fixed in position. The coordinates of Node 0 (source node) and Node *N* (destination node) are (0, 0, 0) and (2500, 2500, 2500), respectively. Thus, the distance between each node will become closer as the number of nodes increases. We perform 2000 trials for each transmitting sound level SL. From Figure 7a, we can see that the transmission redundancy *η* becomes lower with increasing SL, and there are three ways to achieve high data transfer reliability: increasing the transmitting power, sending more redundancies, or reducing the distance between each node. Although increasing the number of nodes is helpful for reducing the packet error rate between each node and thus we can reduce the number of sent packets at each node, the packets in the network may have to travel through more nodes and result in a incremental number of sent packets. Thus, the transmission redundancy *η* does not have significant change when the number of nodes is larger than seven. The average number of packets for transmitting a data packet reduces by about one when SL increases 1 dB. Figure 7b shows the average delay *ϕ* for different number of hops and SL. It can be seen clearly that the average delay for transmitting a packet reduces largely when we increase the number of nodes from four to six. It is because the packet error rate between each node becomes lower, and we could send fewer packets to achieve high throughput. However, when the number of nodes is larger than seven, the average delay is flat or even higher for increased *N*. This is because each node brings a state transition delay (the time for changing state between transmitting and receiving) and each node has to wait for an acknowledgement packet before sending a new block. From the above analysis, we can see that our protocol can work better with a larger number of nodes with the help of nodes’ network coding.

### 5.4. NCHARQ, SDRT, FOCAR, S&W and ARQ Performance Comparison

**Figure 8:**
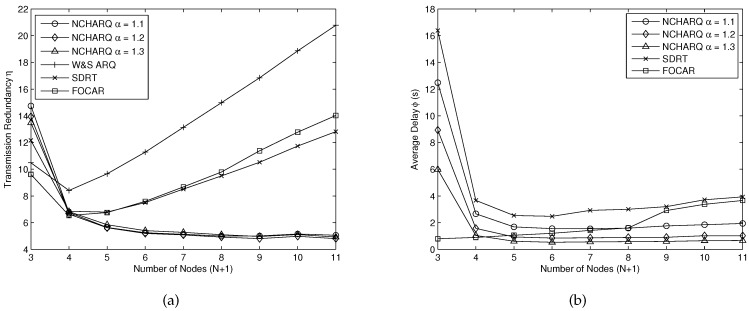
(**a**) the transmission redundancy *η* for different protocols; (**b**) the average delay *ϕ* for different protocols.

Figure 8 shows the performance of SDRT, SDRT, FOCAR, S&W ARQ and NCHARQ protocol. We consider a practical and dynamic scene to conduct our simulations here. We assume that all nodes in the network can move with water flow at the speed of 0–1 m/s. Each node can move in any direction and the structure of the network will change largely with the flow of times. The node distribution is the same as Section 5.3 at the initial state. From Figure 8a, we can see clearly that our proposed protocol NCHARQ can achieve lower transmission redundancy with the help of nodes cooperation, so the improved system can achieve a low energy consumption. When the number of nodes is small (N+1≤4), the distance between adjacent nodes is very long and the transmission links suffer from large packet loss rate. However, for non-cooperative schemes, the increased number of hops may result in a larger number of transmission redundancies because each node has to send a large number of packets to its adjacent downstream node and the node cannot utilize the information from other nodes in the network. For NCHARQ, with the help of all nodes in the network, the transmission redundancy can be much lower when the number of nodes is sufficient. We can save about 50% or more sent packets compared with other protocols when N+1≥7. The redundancy factor *α* does not have a pronounced impact on the performance, but α=1.2 is advisable for it achieves the least *η* in the simulation. Figure 8b shows the average delay *ϕ* for different protocols. When the number of nodes is small, it will be unlikely for nodes to receive packets from other nodes except for the adjacent one. Thus, the network coding will be almost useless and it may be not advisable to use NCHARQ in such a scene. The packet error rate between adjacent nodes is very large ($P_{pac}\left(i,i+1\right)>0.7$ for N+1<4) in this situation, and thus it is suggested to add more nodes to the network to achieve high reliability. It can be seen clearly that our proposed protocol NCHARQ with α=1.2 and α=1.3 achieves the lowest average delay compared with SDRT and FOCAR as the number of nodes increases. For wait and stop ARQ, the average delay is too large (ϕ≥30 s) and is not drawn in the figure. The average delay can be as low as 0.53 s for sending a data packet successfully when N+1=5, which saves at least 50% of the transmission time compared with the other protocols.

## 6. Conclusions

In this paper, we propose a novel reliable data transfer protocol based on network coding and hybrid ARQ (NCHARQ) for underwater acoustic sensor networks. The protocol combines the coding efficiency of network coding and reliability of hybrid ARQ and achieves the asymptotic optimal reliable data transmission in underwater networks. We design a 3D dynamic underwater network model to verify our protocol. Simulation and analysis results show that NCHARQ can save the energy by decreasing the number of sent packets while reducing the average delay. The window estimation algorithm can adaptively change the number of sent packets at each node and thus the protocol can be insensitive to the environment change. For future research, we suggest that a more complex network model and the corresponding relay selection and routing algorithm for our protocol should be investigated. We will also conduct some tests in the real underwater environment in the future.

## Figures and Tables

**Table 1:** The operations in the sender and receiver.

Sender
(1) Updates the sending window size $K_{i}^{\prime }$ for node *i*, encodes the recovered data packets in a block into *N* encoded packets, and sends out $K_{i}^{\prime }$ encoded packets at one time;
(2) Sets a timer and waits for the ACK/NACK feedback. If an ACK is received, then it goes to (1), else goes to (3);
(3) Estimates the retransmission window size *m* with the information from NACK and sends *m* selected encoded packets. Then, it goes back to (2).
**Receiver**
(1) Decodes the received packets from all upstream nodes until no more packets are received from its adjacent upstream node;
(2) Sends an ACK packet back and goes to (3) if the original data in the block can be reconstructed successfully or sends a NACK packet and goes back to (1) if else. The information (lost packets number and index) in an ACK or NACK packet is detailed in Section 4.2;
(3) Encodes the recovered data packets again and transmits them to the next hop.

**Table 2:** Simulation parameters.

Parameter	Value
Maximum mobility range radius *R*	300 m
Sound speed	1500 m/s
Data packet size	50 Bytes
Original data packet number in a block	100
Data rate ($R_{s}$)	10 kbps
Carrier frequency	10 kHz
Synchronous time	10$/R_{s}$
State transition delay	1.5 s
Transmitting sound level	123–125 dB re1μPa @10 kHz
